# Molecular Mechanism of Cyclodextrin Mediated Cholesterol Extraction

**DOI:** 10.1371/journal.pcbi.1002020

**Published:** 2011-03-24

**Authors:** Cesar A. López, Alex H. de Vries, Siewert J. Marrink

**Affiliations:** Groningen Biomolecular Sciences and Biotechnology Institute & Zernike Institute for Advanced Materials, University of Groningen, Groningen, The Netherlands; University of North Carolina, Chapel Hill, United States of America

## Abstract

The depletion of cholesterol from membranes, mediated by *β*-cyclodextrin (*β*-CD) is well known and documented, but the molecular details of this process are largely unknown. Using molecular dynamics simulations, we have been able to study the CD mediated extraction of cholesterol from model membranes, in particular from a pure cholesterol monolayer, at atomic resolution. Our results show that efficient cholesterol extraction depends on the structural distribution of the CDs on the surface of the monolayer. With a suitably oriented dimer, cholesterol is extracted spontaneously on a nanosecond time scale. Additional free energy calculations reveal that the CDs have a strong affinity to bind to the membrane surface, and, by doing so, destabilize the local packing of cholesterol molecules making their extraction favorable. Our results have implications for the interpretation of experimental measurements, and may help in the rational design of efficient CD based nano-carriers.

## Author Summary

The ability of certain molecules to capture other molecules forming so-called inclusion complexes has a range of potential important applications in e.g. drug delivery and chemical sensing. Here we study the complexation of cholesterol by small oligosaccharide rings named cyclodextrins (CDs). Cholesterol is an essential lipid in the plasma cell membrane, and the ability of CDs to extract cholesterol is widely used in the biomedical field to control the level of cholesterol in the membrane. The molecular mechanism of this process, however, is still not resolved. Using a detailed computational model of cholesterol and CD, we have succeeded to simulate this extraction process. We observe that the CDs are rapidly binding to the membrane surface in a dimeric form, and, provided that the CD dimers are in a suitable orientation, cholesterol molecules are being extracted spontaneously. The cholesterol/CD inclusion complex remains adsorbed on the surface; our simulations predict that the rate limiting step for the actual transport of cholesterol is the desorption of the complex from the membrane. With a clearer understanding of the basic molecular mechanism of the CD mediated process of cholesterol extraction, we can begin to rationalize the design of more efficient CDs in numerous applications.

## Introduction

Among all cyclodextrins (CDs), the most abundant are 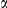-, 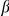-, and 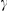-CDs with six, seven and eight glucopyranose monomers, respectively. They have a rigid conical molecular structure with a hydrophobic interior and a hydrophilic exterior. The internal cavity of these molecules is able to include a wide range of guest molecules, ranging from polar compounds such as alcohols, acids, amines, and small inorganic anions, to non-polar compounds such as aliphatic and aromatic hydrocarbons, while the hydrophilic exterior helps CDs to interact favourably with water. Due to the structural simplicity and small size of CDs, combined with negligible cytotoxicity, they are considered as very suitable nano-delivery vehicles [1]–[4]. They may be combined into larger assemblies such as polymeric networks or nanoparticles, and used for controlled drug-delivery, chemical sensing, or as excipients for a large diversity of compounds [5]–[10] with applications in fields ranging from food technology, pharmacology, and cosmetics to environmental chemistry.

Another important application is the use of cyclodextrins to manipulate lipid composition in different cells. Numerous studies have shown that exposing cells or model membranes to CDs results in removal of cellular cholesterol [11]–[17]. The degree of cholesterol depletion is a function of the CD derivative used, its concentration, incubation time, temperature and cell type. In particular 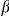-cyclodextrin (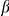-CD) has been shown to be the most efficient sterol-acceptor molecule, apparently due to the diameter of its internal cavity that matches the size of these molecules [18], [19]. The question how CDs are able to remove cholesterol is open to discussion. Originally the idea was proposed that CD remains in the aqueous phase, stabilising the monomer population during the naturally occurring exchange of lipids from the membrane to the aqueous phase [20]. More recently, several authors support the desorption model [2], [11], [16], [21]–[23], in which cyclodextrins interact directly with membrane embedded-cholesterol. Yancey et al. [16] proposed that cyclodextrin molecules are able to diffuse into the proximity of the plasma membrane, so cholesterol molecules could enter directly into the hydrophobic pocket of the cyclodextrin, without the necessity of completely desorbing through the aqueous phase. Mascetti et al. [14] proposed a model based on polarization modulation infrared absorption spectroscopy (PMIRRAS) and Brewster angle microscopy (BAM), supported by ab-initio calculations, in which 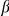-CD molecules stack parallel to the plane of the membrane and form perfect channel structures in direct contact with cholesterol monolayers.

Despite the substantial amount of experimental effort, the molecular mechanism by which cholesterol is removed remains unclear. The Molecular Dynamics (MD) technique provides a suitable tool to investigate this process at atomistic resolution. Previously, CDs have been simulated in aqueous environment, providing structural, dynamic and energetic information of CD aggregates and various inclusion complexes [23]–[27]. The interaction of CDs with membranes has not been addressed thus far in computational studies. Here, we use MD simulations to study the 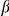-CD mediated extraction of cholesterol from model membranes, in particular from cholesterol monolayers. Experiments with monolayers have shown that cholesterol can be efficiently removed [16]. Our results show that efficient cholesterol removal requires the presence of 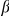-CD dimers, which need to be oriented perpendicular to the membrane surface. Both requirements are favoured at high CD concentration. Based on our results we propose a molecular model for the extraction of cholesterol from membranes, with detailed free energy estimates of the key intermediate steps.

## Results/Discussion

In this section, we first discuss results obtained from systems in which CD dimers were placed in direct contact with the monolayer surface, in an upright conformation. Under these conditions the uptake of cholesterol occurs readily. We proceed with results obtained with other systems with less biased initial configurations, showing that efficient extraction of cholesterol indeed requires a CD dimer as well as a suitable orientation of the dimer with respect to the monolayer surface. Then we present results from our free energy calculations to quantify the energetics of the extraction process. In the final part, a molecular model for CD mediated cholesterol extraction is presented based on the combined data from our simulations and the existing experimental knowledge.

### Cyclodextrins in action

**Figure 1:**
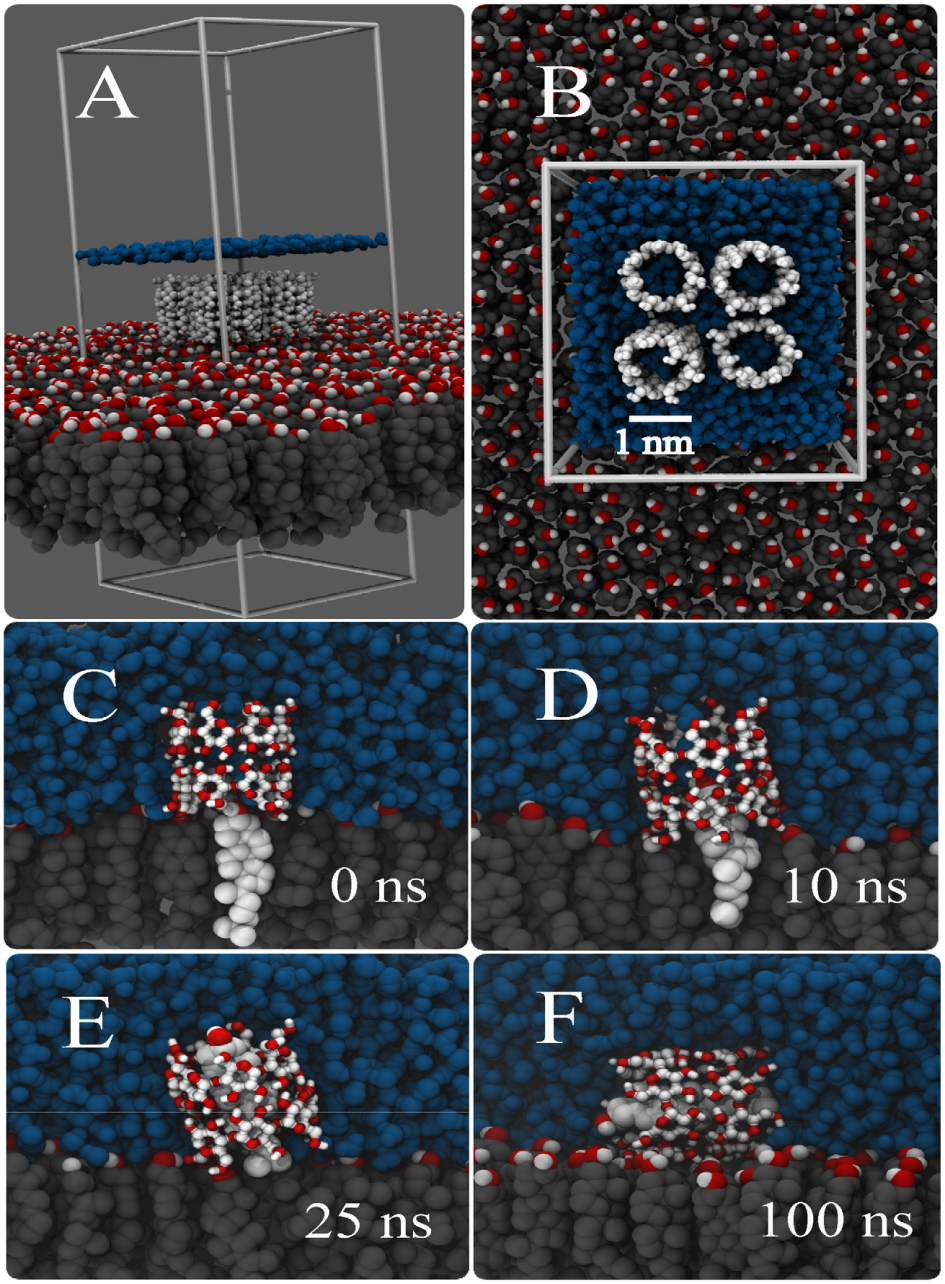
Spontaneous extraction of cholesterol by a cyclodextrin dimer. Panels (A,B) show the initial system set-up with four CD dimers sitting on top of a pure cholesterol monolayer, panels C–F show the time evolution of the extraction of cholesterol by one of the CD dimers. At 0 ns the cholesterol is still inside the monolayer (C), at 10 ns the upper part is sucked in (D), and after 25 ns the cholesterol is almost fully inside apart from the tail (E). Tilting of the complex after 100 ns completes the extraction process (F). Color code; cholesterol body: grey, cholesterol head: red-white, CDs: white and red, water: blue (In panel A the level of the water layer is only depicted by a blue line).

A series of snapshots from a typical cholesterol extraction simulation is depicted in Figure 1. Our set-up consists of a pure cholesterol monolayer at temperature and pressure conditions which proved to be optimal for cholesterol extraction in-vitro [15] (see Methods). Based on experimental evidence, we assumed that the inclusion complex would consist of a 2∶1 CD∶cholesterol stoichiometry [18], [28], [29]. Initially, four 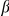-CD dimers were placed close to the surface of the cholesterol monolayer (Figure 1A–B). In the simulation, the 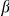-CDs rapidly bind to the cholesterol monolayer interface. During the 200 ns simulation, each of the four dimers extracts a cholesterol molecule from the membrane. The extraction process is illustrated in Figure 1C–F for one of these dimers. Soon after the binding of the 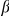-CD dimer to the interface (Figure 1C), in about 10 ns, there is the imminent immersion of cholesterol into the hydrophobic channel (Figure 1D). Within 25 ns, the cholesterol is sucked in further to the point that its hydroxyl head group sticks out at the other side of the nano-channel, into the aqueous phase (Figure 1E). Although the cholesterol is embedded quite deeply within the channel, the dimethyl end of its hydrophobic tail is still in contact with the surface of the monolayer. It is not until the CD/cholesterol complex tilts by 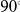 that the cholesterol becomes completely desorbed from the monolayer (Figure 1F). The tilting takes place after 100 ns, exposing the less polar part of the 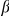-CD (i.e. the ring of every glucose monomer) directly to the surface of the monolayer and allowing hydroxyl groups of 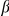-CD to hydrogen-bond with the hydroxyl groups of adjacent cholesterol molecules. This conformation remains stable until the end of the simulation time, at 200 ns. The time required to completely extract the cholesterol from the monolayer varied between the individual dimers from 20 to more than 100 ns, with the rate limiting step the tilting of the complex. We set up five independent simulations under the same conditions, obtaining similar behavior for every case. From our simulations we can make a crude estimate of the cholesterol extraction rate. With four cholesterols extracted on a 100 ns time scale from a patch of 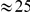 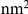, we obtain a rate of 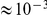 molecules 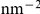 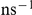. This is much faster than the reported desorption rate of 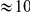 pmol 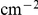 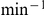 [15], corresponding to 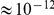 molecules 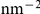 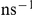. Although part of the discrepancy may be attributed to the difference in concentration (our simulations were performed at an overall concentration of 0.1 M, but the equilibrium concentration in the aqueous sub-phase is unknown), we conclude that the experiments probe the actual desorption of the CD/cholesterol complex from the monolayer surface. This requires a much longer time scale, involving a large free energy barrier as will be discussed later. Concentrating for now on the actual formation of the complex, our simulations suggest that cholesterol extraction is favoured by two conditions: (i) the stability of the dimer on the monolayer, and (ii) the orientation of this dimer with respect to the cholesterol molecules. These conditions are discussed in more detail next.

### Efficient extraction of cholesterol requires suitably oriented cyclodextrin dimer

We observed that the successful extraction of cholesterol was linked directly to the stability of the 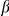-CD dimer. Simulations in which the dimer stability was decreased, using dimers in a head-tail or tail-tail orientation, resulted in formation of monomers; in their monomeric form cholesterol extraction is not observed on the time scale of our simulations (see Text S1 for details, Figure S1 and S2). Although the 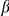-CD monomers keep interacting with the cholesterol monolayer (Figure S2), the interaction strength between a single ring and cholesterol is apparently not enough to extract it. The stoichiometry for the 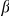-CD-cholesterol complex is still under debate, since it is highly dependent on the conditions used in the experiment (e.g. dielectric constant of the solvent, salting conditions, concentration of the molecules). Ravichandran et al. [18], based on NMR and UV data, concluded a preferential 2∶1 stoichiometry for 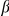-CD and cholesterol. Williams et al. [29] found stoichiometry ratios for the hydroxypropyl 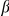-CD (HP-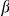-CD)/cholesterol complex changing from a 1∶1 to 2∶1 ratio on increasing concentration of the carbohydrate. By a different approach, Tsamaloukas et. al. [28] concluded the preference for a 2∶1 stoichiometry for randomly methylated 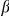-CD with cholesterol in the presence of lipid vesicles. Our results are consistent with this preferred 2∶1 stoichiometry, as the 1∶1 complex is not formed spontaneously at least in the presence of a cholesterol monolayer. Further below we will show results from free energy calculations that support the 2∶1 stoichiometry, also under pure aqueous conditions.

**Figure 2:**
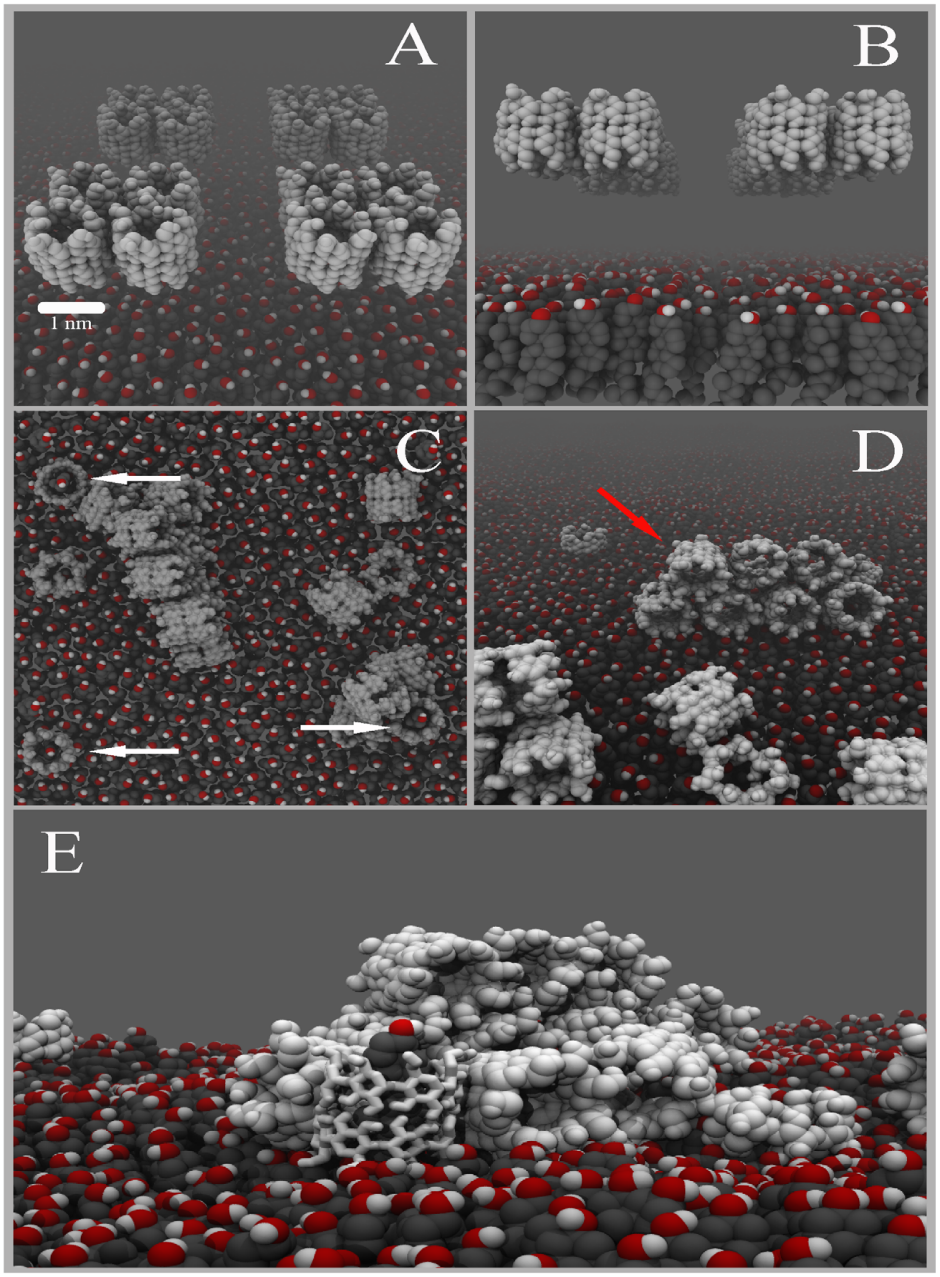
Collective binding of cyclodextrins and subsequent cholesterol extraction. Panels (A,B) show the initial system set-up with 16 CD dimers in aqueous solution next to a cholesterol monolayer. Panels (C,D) show the final configuration of the systems after 200 ns. The CDs adopt a number of configurations, either stacked (red arrow), or as single layer in either a tilted or untilted orientation with respect to the surface normal. Dimers present in the untilted orientation (white arrows) are capable of extraction cholesterol. A close up of a CD/cholesterol complex, stabilized in an upright position by a cluster of stacked CDs, is shown in panel E. Color code; cholesterol body: dark grey, cholesterol head: red-white, CDs: light grey. Water is not depicted for clarity.

The ability to uptake cholesterol from the monolayer also depends on the orientation of the dimer with respect to the surface. To explore this more systematically, we performed additional simulations in which single dimers or pairs of dimers were placed on the cholesterol monolayer. When a single dimer is placed in direct contact with the surface of the monolayer, it is unable to keep a straight conformation, tilting immediately by 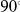 and remaining in this position for the rest of the simulation time (Figure S2). On the contrary, a pair of dimers is able to keep a straight conformation long enough to allow the cholesterol to enter the hydrophobic cavity of either one or both of the dimers, ending with an effective extraction of cholesterol. With four dimers present, the process is even more efficient as we showed in Figure 1. Thus, the efficient desorption of cholesterol appears to be a cooperative process between several 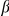-CD dimers. To verify the importance of orientation and cooperative effects, we increased the system size to a monolayer of 252 cholesterols interacting with 16 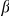-CD dimers (Figure 2A). To remove the initial bias of having the dimers already close to the interface, here we started with dimers placed further away in solution (Figure 2B). We observe that nearly all of dimers end up binding to the monolayer surface. The time scale of binding varies between 50 and 150 ns, governed by the random diffusion of the monomers. When they approach the surface to within 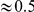 nm, they bind irreversibly. We also note that the dimers are stable and do not dissociate into monomers either in solution or when adsorbed on the monolayer. We further observe that the 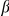-CDs aggregate on the monolayer forming stacked barrels (Figure 2 C,D), mostly tilted by 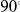 with respect to the monolayer surface normal. Some, however, are attached in the correct conformation for the extraction process to occur (Figure 2 C,E indicated by arrows), stabilised in this position by adjacent CDs. The same qualitative behaviour was observed in three independent simulations. The observation of multiple layers of CDs stacked on top of each other seems to be in good agreement with BAM experiments showing 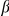-CDs interacting with monolayers and forming bodies of different heights [14]. However, based on ab-initio calculations to interpret their spectroscopic measurements, Mascetti et al. concluded a perpendicular stacking of the CDs. We clearly observe tilted layers (Video S1), which corroborates with experimental data for the adsorption of CD at the water-air interface with highly concentrated CD solutions [25]–[30].

### Energetics of cholesterol desorption

**Figure 3:**
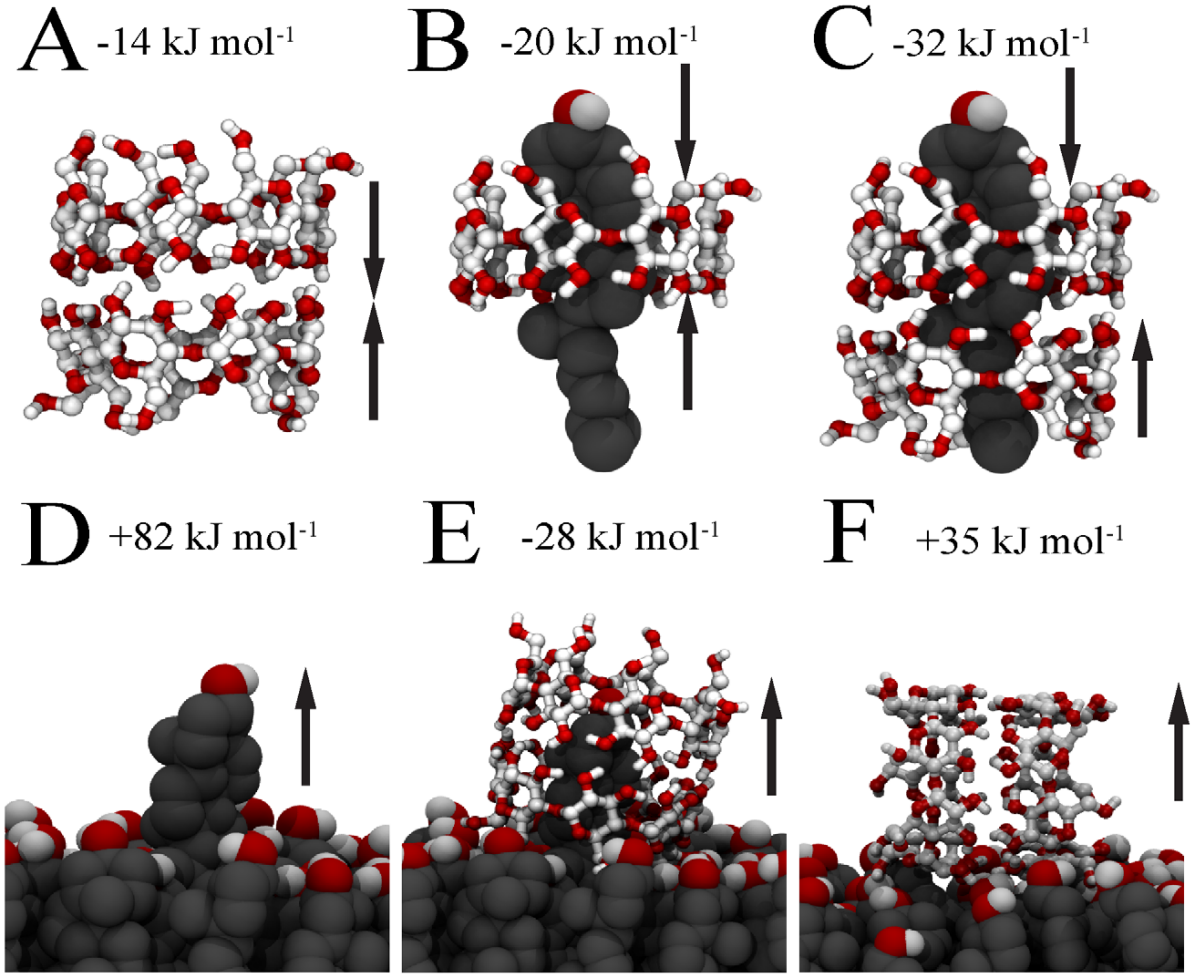
Free energies of sub-steps of the cholesterol desorption process. (A) Association of CD dimer, (B) Binding of cholesterol to a CD monomer, (C) Binding of cholesterol to a CD dimer, (D) Extraction of cholesterol from cholesterol monolayer, (E) Extraction of cholesterol mediated by a 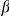-CD dimer, (F) Desorption of tilted CD dimer from cholesterol monolayer. The values of the associated free energies are given for each sub-step.

Our results show that cholesterol extraction is energetically favorable, especially by a CD dimer, but also that tilting and clustering of CDs plays an important role. To understand in more detail the energetics of the whole process, we have considered several sub-processes and calculated the associated free energy changes through calculations of potential of mean force (PMFs) along reaction paths (see the Methods section). An overview of the sub-processes and calculated free energies is given in Figure 3. First, we calculated the dissociation free energy for a CD dimer in water (Figure 3A). The resulting free energy is 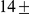
2 kJ 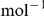, implying a clear stabilization of the dimeric conformation, in line with proposed aggregation models based on experimental evidence [31]. Note that the two monomers can bind in three different relative orientations, namely head-head, head-tail and tail-tail. The head-tail as well as the tail-tail orientations were found to be significantly less stable, as these dimers spontaneously dissociate in water (see Text S1). Next, we looked at the binding free energy of cholesterol inside either a CD monomer or dimer with respect to the aqueous phase (Figure 3B,C). The driving force for the formation of these inclusion complexes is believed to arise from a combination of non-covalent interactions such as van der Waals forces (hydrophobic interior), electronic effects (probably due to the presence of hydroxyl groups in the glucose rings), and steric factors (the volume size of the hydrophobic cyclodextrin cavity) [32]. In the case of the monomer, we find that the binding free energy equals 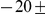
3 kJ 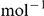, while for the dimer this energy is 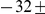
5 kJ 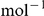. The binding of cholesterol to CD is thus favourable in each case, but significantly more so with respect to the dimer. Experimentally it is difficult to distinguish between the 2∶1 and 1∶1 complexes, and different methods predict binding constants varying over orders of magnitude. Keeping these limitations in mind, a comparison of the binding constants calculated from our PMFs predict an order of magnitude comparable to the experimental estimate [33], [34] for 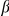-CD and HP-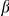-CD, assuming the experiments probe the 2∶1 stoichiometry. In experiments on DM-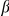-CD [35], the binding affinity could be differentiated between the 2∶1 and 1∶1 stoichiometries; the 2∶1 case showed a much higher affinity in line with our results for 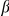-CD (See Text S1 for details)

**Figure 4:**
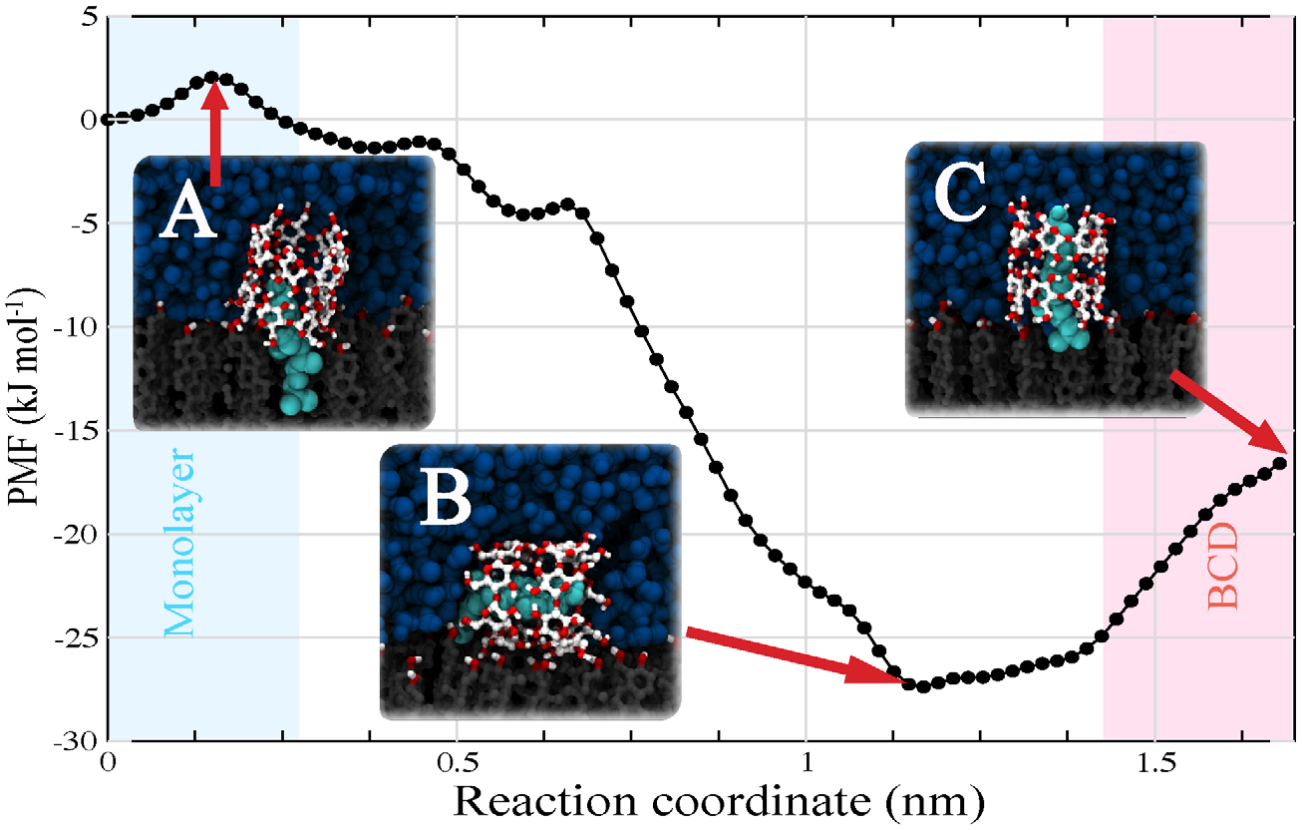
Potential of mean force for CD mediated cholesterol extraction. Using the umbrella sampling method, cholesterol was slowly extracted into a CD dimer adsorbed on the cholesterol monolayer. The reaction coordinate represents the distance between the centers of mass of the cholesterol and the cholesterol monolayer. A small activation barrier at the beginning of the process is noticeable (state A). As the reaction coordinate increases, the free energy is lowered by the encapsulation of cholesterol inside the CD dimer. Beyond a distance of 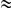
0.7 nm, the energy drops even further due to the tilting of the whole complex. The lowest free energy (−28 kJ 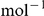) is found for the complex laying parallel to the surface (state B). Pulling cholesterol further away from the monolayer forces the complex back toward an upright orientation (state C) with an associated increase of the free energy by 11 kJ 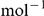. The energy difference between state A and C, corresponding to the direct extraction of cholesterol, is −17 kJ 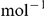.

The binding of cholesterol to the cholesterol monolayer was also considered (Figure 3D). The energy needed to extract a single cholesterol molecule completely from the monolayer to the water bulk is found to be 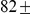
2 kJ 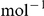. This value is similar to the values reported for the binding free energy of cholesterol in membranes of different lipid mixtures [36], ranging from 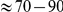 kJ 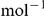 depending on lipid composition. However, it is more than twice the energy required to extract it from a 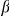-CD dimer. This leads to the apparent conclusion that the energy penalty to extract cholesterol from the monolayer (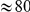 kJ 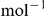 cost) cannot be provided by embedding it inside cyclodextrin (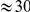 kJ 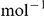 gain). Yet we showed that cholesterol is spontaneously extracted from the monolayer when CDs adsorb on the surface (cf. Figure 1 and 2). To shed further light on this, we also computed the free energy profile for the extraction of cholesterol into a monolayer-adsorbed 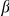-CD dimer. The result is shown in Figure 4. The process is clearly downhill in free energy, with a stabilization of 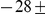
3 kJ 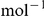 for the formation of the CD/cholesterol complex. During the free energy calculation, we observed spontaneous tilting of the complex as soon as the rigid body of cholesterol was extracted, in line with our previous results. Restraining the complex to an upright orientation, the free energy is increased by 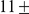
2 kJ 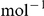, but the overall free energy for extraction is still favorable. These results suggest that it is not only the interaction between the cholesterol and the inner core of 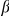-CDs but also the disrupting effect of the carbohydrate on the water-monolayer interface which drives the complexation between cholesterol and cyclodextrin. Put differently, the binding of CD to the monolayer locally disrupts the packing of cholesterol, favouring the uptake of cholesterol. To complete our free energy analysis, we therefore computed the binding free energy of the empty CD dimer to the interface, starting from a tilted conformation (Figure 3F). As expected from our simulations (cf. Figure 1 and 2) binding of cyclodextrin to the monolayer is favorable, with the tilted configuration more favorable than the straight one (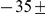
4 kJ 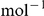 versus 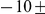
2 kJ 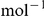) (See also Figure S3). The high affinity of sugars for membranes is also exemplified by cryo- and anhydro-protective properties of many sugars, stabilizing membranes in low temperature or dehydrated states [37].

### Molecular model for cyclodextrin mediated cholesterol extraction

**Figure 5:**
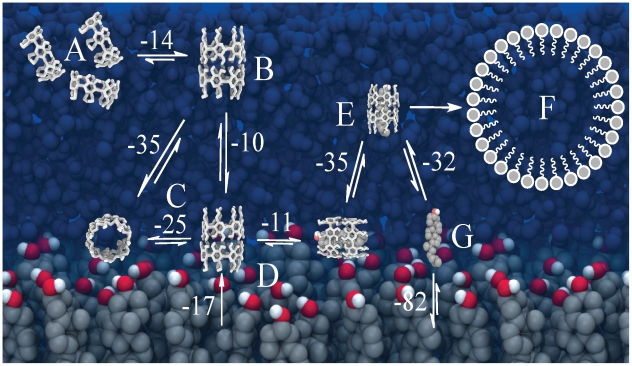
Molecular model for cyclodextrin mediated cholesterol extraction. (A) Association of CDs in aqueous solution leads to the formation of dimers (B), which bind to the membrane surface assuming either a tilted or untilted configuration (C). The latter configuration is suitable to extract cholesterol, allowing the formation of a membrane-bound complex (D). Desorption of the complex brings cholesterol in solution (E), facilitating its transfer to other membranes (F). Direct desorption of cholesterol is energetically much more costly (G) (Energy values are in kJ 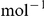).

**Figure 6:**
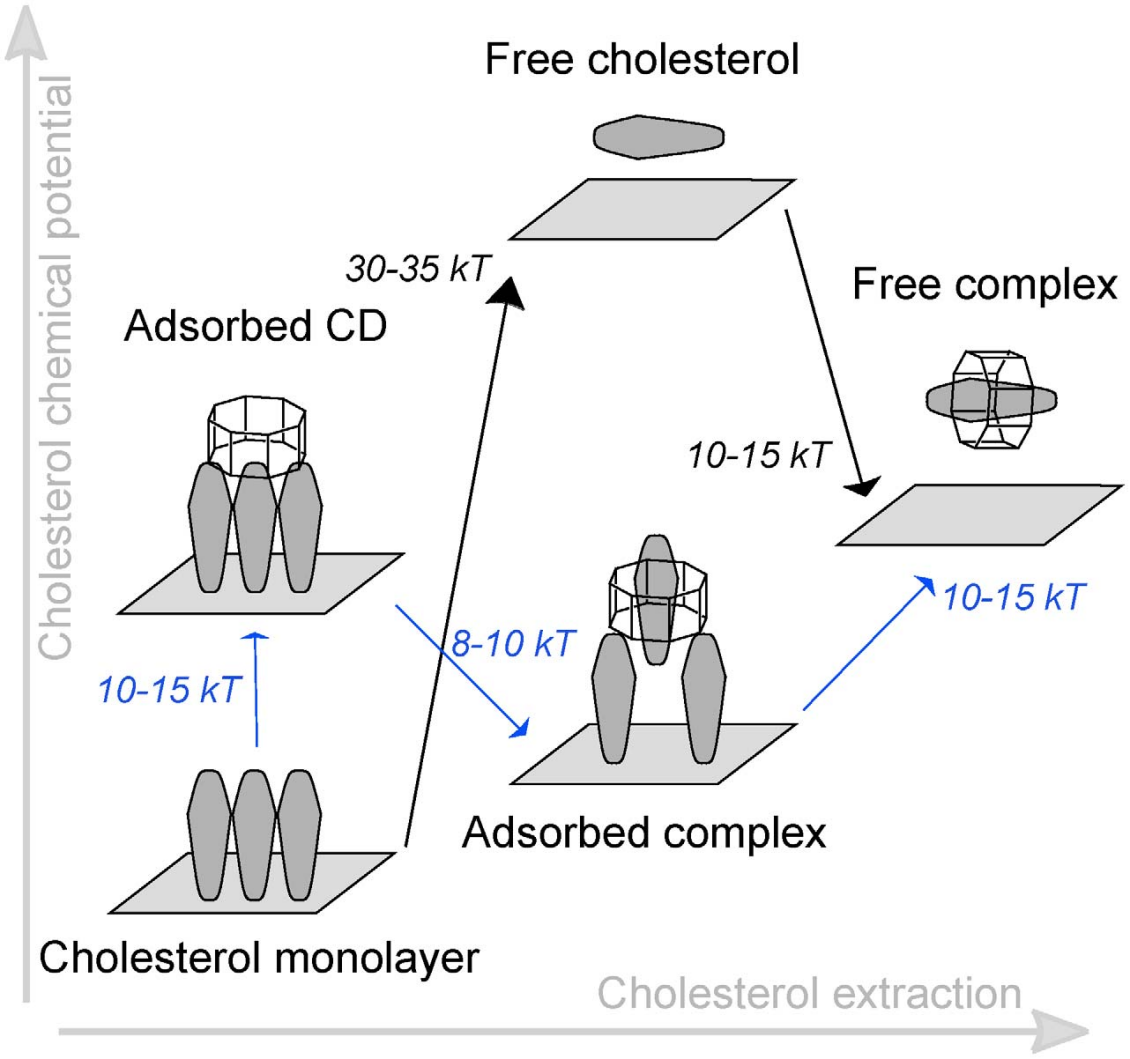
Comparison of surface mediated and solvent mediated cholesterol-CD complexation. Schematic overview of the energetic steps involved in the unassisted desolvation of cholesterol (black) versus the CD mediated desolvation (blue) of cholesterol from the membrane. The unassisted pathway involves a large energy barrier of 30–35 kT to put cholesterol in solution before it complexes with CD, whereas the surface mediated route only involves a barrier of 10–15 kT required to dissociate the cholesterol/CD complex from the membrane surface.

Based on the results of our simulations, combined with the current body of experimental results, we propose a molecular model for the process of cholesterol extraction by 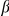-CDs. The model is shown in Figure 5. When 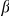-CDs are in solution (Figure 5A), they have a strong tendency to aggregate; depending on overall concentration, the equilibrium will shift away from monomers to dimers (Figure 5B) or higher order aggregates. In the presence of a membrane surface, the cyclodextrins will bind, adopting a tilted conformation (Figure 5C). Tilted structures are not able to extract cholesterol easily, but due to their surface activity the amount of cyclodextrins bound to the membrane accumulates. A high density of CDs increases the probability of having straight conformers in addition to barrel like structures. The straight orientation is optimal for the cholesterol extraction, which is a downhill process at this point (Figure 5D) due to the destabilization of cholesterol packing underneath the cyclodextrins. Desorption of the complex from the membrane surface, however, is associated with a large free energy barrier. Occasionally it will occur, leading to the formation of complexes in solution (Figure 5E). Since there is a small energy difference between the 2∶1 and 1∶1 complexes, the relative population will depend on the concentration of 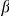-CD. Finally, once desorbed, cholesterol molecules could be transferred to e.g. lipid vesicles or lipoprotein particles (Figure 5F) by a simple diffusion mechanism. In Figure 6 we further compare the two possible mechanisms by which the CD/cholesterol complex can be formed: either via desolvation of cholesterol into the aqueous phase (‘solvent mediated’) or via the desorption from the monolayer directly into cyclodextrin (‘surface mediated’). Direct desorption of cholesterol costs 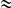
80 kJ 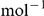 (Figure 5G), thus making CD mediated extraction much more efficient (requiring only 35 kJ 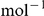 to dissociate the dimer from the surface, assuming a tilted configuration). The 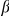-CD-monolayer interaction decreases the cholesterol-monolayer stability, lowering the energetic barrier for cholesterol desorption (Figure 6, blue lines). Our energetic analysis is consistent with estimates from experiments on different cell types and model membranes [16], reported as 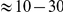 kJ 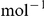 for CD mediated transfer (depending on cell type) and 84 kJ 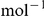 for direct transfer of cholesterol.

At this point we discuss the relevance of our model to the interpretation of experiments performed under physiological conditions. Our simulations concern pure cholesterol monolayers only, and a CD concentration around 0.1 M. These conditions were chosen because they are optimal for rapid cholesterol extraction in our simulations, allowing the process to be studied on the nanosecond time scale that is accessible to atomistically detailed simulations. Compared to the experimentally measured desorption rate of 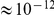 molecules 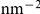 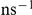, the rate of desorption in our simulations is about 9 orders of magnitude faster (see ‘Cyclodextrins in action’ section). We attribute this difference to the different measures of the desorption rate in experiment versus our simulations. Experiments calculate desorption rates by means of the area per lipid change on monolayers (see Ohvo et al. [15]), and are therefore sensitive to the desorption of the complex from the monolayer. In our simulations, however, we measure only the rate of extraction of cholesterol into the CD complex. According to our results, the energy 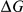 for the complex desorption is about 14 kT; assuming the kinetics of the process scales with 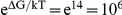, we can already account for 6 orders of magnitude in the difference in the desorption rate. Other energy barriers that might affect the experimental rate of desorption are the tilting/untilting of the complex, or the formation of dimers at the interface. Dissociation of cholesterol from the complex in water could, in principle, also pose another barrier. However, in Ohvo et al. [15], cholesterol desorption rates were also measured by taking aliquots of the sub phase and measuring the amount of radio-active labelled cholesterol. The same desorption rate was found as with the other approach based on changes in monolayer area, indicating that it is likely that the measured cholesterol in the aqueous sub phase is still complexed to CD. In addition, CD concentration may play an important role in the details and rate of the extraction process. Experimentally, typical concentrations are in the 1–10 mM range. The effective concentration of CDs at the membrane surface is predicted to be orders of magnitude higher, based on the 35 kJ 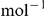 adsorption energy we obtained from our simulations. As we do not observe spontaneous exchange of CDs between the adsorbed and dissolved states in our simulations, we cannot assess the equilibrium concentration of CDs in the aqueous phase, making a direct comparison toward experiment impossible in this respect. A high surface concentration of CDs is likely to facilitate the cholesterol uptake in two manners. First, the propensity to form dimers, which are more efficient in binding cholesterol compared to the monomers increases. Second, uptake of cholesterol from the membrane requires an upright position of CD, which is stabilized at high CD concentration. Based on our results one predicts a cross-over from monomer mediated cholesterol extraction at low CD concentration toward dimer mediated extraction at higher concentrations, and possibly even higher order aggregates at further increase of the CD concentration. The point at which the cross-over takes place will be highly dependent on the composition of the membrane, which may act in multiple ways in affecting the cholesterol extraction process. The most direct way is by stabilizing cholesterol, e.g. inside raft-domains, or destabilizing it in membranes composed of poly-unsaturated lipids. Indirectly, the membrane composition will play a role in the efficiency of the carbohydrates to bind to the membrane surface. Probing the interplay between lipids, cholesterol and cyclodextrins is currently being investigated. Some of the conflicting experimental data indicating either 1∶1 or 2∶1 complexes might result from this non-trivial concentration dependency.

In summary, using atomistically detailed simulations, we were able to reveal the molecular mechanism of how cholesterol is extracted by 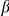-CDs from a cholesterol monolayer. From our results we conclude that the desorption involves a number of sub-steps: i) formation of CD dimers, ii) binding of CDs at the interface, iii) adsorption of cholesterol into CD, iv) tilting of CD, v) desorption of CD/cholesterol complex from the interface. Only the last step involves a substantial energy barrier, the other processes are essentially downhill. However, depending on the overall concentration of CDs the tilting of CD might take place before the cholesterol uptake, leading to a potential second kinetic barrier. With a detailed understanding of the basic molecular mechanism of this process we can begin to rationalize the design of more efficient CDs in numerous applications.

## Methods

### System setup

We simulated systems of different size consisting of a pre-equilibrated cholesterol monolayer, 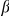-CD dimers and water molecules. The initial coordinates of 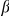-CD were taken from the crystal structure [19]. For the small system, the cholesterol monolayer consisted of 52 molecules, with a lateral area of 24.7 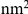. Four 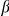-CD dimers in head-head, head-tail or tail-tail conformation were placed with the hydroxyl groups in direct contact with the monolayer and solvated by 1,800 water molecules (2.5 nm water layer). Periodic boundary conditions were applied in all directions. In the direction perpendicular to the monolayer, a vacuum layer of 3.0 nm was added in order to avoid direct interaction between mirror water molecules and the tails of cholesterol in the monolayer. A snapshot of the initial conditions is depicted in Figure 1A. The big system was prepared similarly, and consisted of 252 cholesterol molecules, 16 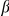-CD dimers, and 13,400 water molecules. Here, the 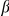-CD dimers, were initially placed at a distance of 1.0 nm away from the monolayer surface (cf. Figure 2A,B). To avoid the interaction between cholesterol tails and mirror waters, a vacuum slab of 4.0 nm was added. The cyclodextrin concentration was 0.2 M for the small and 0.1 M for the big system. For the free energy calculations, additional systems were set up with only a single cyclodextrin/cholesterol complex in excess water, or a cholesterol monolayer with one cyclodextrin monomer or dimer adsorbed.

### Simulation details

Simulations were performed using the GROMACS molecular dynamics package [38]. The parameter set for the simulation of 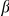-CD was taken from the latest Gromos force field for carbohydrates [39]. The parameters for cholesterol were taken from previous work done by Höltje et al. [40]. The SPC water model [41] was used to model the solvent. A 2 fs time step was used to integrate Newton's equations of motion. The LINCS algorithm [42] was applied to constrain all bond lengths. Non-bonded interactions were handled using a twin-range cut-off scheme [43]. Within a short-range cut-off of 0.9 nm, the interactions were evaluated every time step based on a pair list recalculated every 5 time steps. The intermediate range interactions up to a long-range cut-off radius of 1.4 nm were evaluated simultaneously with each pair list update, and assumed constant in between. To account for electrostatic interactions beyond the long-range cut-off radius, a reaction field approach [44] was used with a relative dielectric permittivity of 68. The temperature was maintained at 288 K by weak coupling of the solvent and solute separately to a Berendsen heat bath [45] with relaxation time of 0.1 ps. The pressure of the systems was controlled also by weak coupling, with a relaxation time of 1 ps. An anisotropic coupling scheme was used to maintain a constant surface pressure of 33 mN 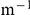 (See Text S1 for details about the computation of the surface pressure). At this pressure the area per lipid equals 48 Å^2^ in our simulations, in very good agreement with experiments [14]. Notice that the conditions (temperature as well as surface pressure) used here have been previously reported to be optimal for cyclodextrin mediated cholesterol desorption from cholesterol monolayers [15]. Before production time, the systems were pre-equilibrated by slow heating up to 288 K. Multiple simulations were performed starting from randomized initial velocities. In total five independent simulations of 200 ns for the small system, and 3 simulations of 200 ns for the big system were performed.

### Free energy calculations

An umbrella sampling approach was used to calculate the potential of mean force (PMF) for a number of important sub-steps related to the total desorption process, namely i) the extraction of one single cholesterol molecule from the monolayer, ii) extraction of cholesterol from a single 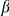-CD ring or from a 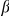-CD dimer, iii) dissociation of the 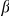-CD dimer, and iv) desorption of the 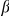-CD monomer and dimer from the cholesterol monolayer. For the PMF calculations, we used the umbrella sampling method [46] with 18 window points, spaced by 1 Å, restraining the center of mass of one cholesterol with respect to the center of mass of the monolayer (i), a 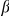-CD monomer or dimer (ii), or between the two monomers (iii), or between the cyclodextrin and the monolayer (iv). The restraining potential was harmonic with a force constant of 1,000 kJ 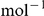 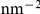. 50 ns of simulation was performed for each window, covering a total of 0.9 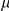
s per system. The PMFs were reconstructed using the weighted histogram analysis method [47], with 200 bins for each profile. To estimate the convergence in the PMF, each window trajectory was divided in blocks. The statistical error was calculated from the variance between averages over individual blocks, using a block averaging procedure. Blocks were found to be statistically independent over 1–5 ns time intervals. In the case of the complex formation between cholesterol and cyclodextrin, the equilibrium binding constant 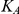 (Table S1) can be calculated within the framework of classical statistical mechanics using the following expression [24], [48], at 1 M standard state:
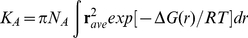
(1)where 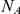 is Avogadro's number and 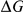 is the calculated PMF as a function of the distance 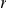 between the centers of mass of cholesterol with respect to CD. 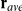 is the average radius of the cross section of CD to which the cholesterol is confined, and depends on the reaction coordinate 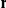. From the expression of 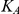, the association or binding free energy 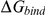 may be obtained:
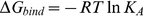
(2)


## Supporting Information

Figure S1Stability of 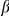
CD dimers. Panel A shows the initial dimeric conformations for head-head, head-tail or tail-tail oriented CDs, respectively. Numbering of the carbon atoms in the monomer is given in panel B. In panel C, the number of inter-monomer hydrogen bonds as a function of simulation time is shown for the three different conformers.(EPS)

Figure S2Ineffective cholesterol extraction. Four 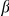
CD dimers in head-tail orientation were added in direct contact with a cholesterol monolayer (A). However, during the simulation, the structure is completely disrupted, leaving only monomers interacting with the head of cholesterols (B,C). Single dimers in a head-head orientation are stable (D), but are not effective for cholesterol extraction, since they prefer a tilted conformation (E).(EPS)

Figure S3 Potentials of mean force of key steps of cholesterol extraction. (A) Extraction of cholesterol from CD monomer, (B) extraction of cholesterol from CD dimer, (C) desorption of CD dimer from cholesterol monolayer, and (D) desorption of cholesterol from monolayer. The potentials are shown as a function of the reaction coordinate being the distance between the centers of mass of the extracted molecule and the host system.
(EPS)

Table S1Association constants of cholesterol with CDs. Association constants K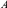 of cholesterol and 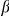-Cyclodextrin (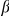-CD), Hydroxypropyl-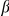-Cyclodextrin (HP-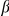-CD) and Di-Methyl-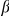-Cyclodextrin (DM-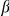-CD), calculated from our simulations and compared to experiments (a, b and c were taken from references [33], [34] and [35] respectively.(EPS)

Text S1 Details about the free energy calculations, and results for control simulations.
(PDF)

Video S1 Simulation of cholesterol extraction from a cholesterol monolayer by cyclodextrins. Initially, CD dimers are in the aqueous phase above the cholesterol monolayer. As the simulation proceeds, the CDs bind to the membrane surface and form aggregates of different kinds. Suitably oriented CD dimers are able to extract cholesterol from the monolayer. The CD dimers are shown in white, cholesterol in grey with red hydroxyl groups. The extracted cholesterol molecules are highlighted in purple. Water is shown transparently.
(MPG)
