# Supplementary material for: Molecular Mechanism of Cyclodextrin Mediated Cholesterol Extraction
Source: PLoS Comput Biol. 2011 Mar 24;7(3):e1002020. doi: 10.1371/journal.pcbi.1002020 (PMC3063748; doi:10.1371/journal.pcbi.1002020)
Supplement: Text S1 — Details about the free energy calculations, and results for control simulations. (PDF) [file pcbi.1002020.s005.pdf]

"Molecular mechanism of cyclodextrin mediated cholesterol extraction"

Cesar A. López, Alex H. de Vries, Siewert J. Marrink

Supporting information

SUPPLEMENTARY METHODS

Surface pressure calculation of cholesterol monolayers

The surface tension in GROMACS [1] is calculated from the diagonal components of the pressure tensor:

$$\gamma_s = h_z \left(P_{zz} - \left(\frac{P_{xx} + P_{yy}}{2} \right) \right) \tag{1}$$

where h_z is the z-component of the box size. The quantity P_{zz} is the pressure normal to the monolayer and $\frac{P_{xx}+P_{yy}}{2}$ is the pressure tangential to the monolayer, therefore the surface tension is the difference between the normal and lateral pressures on the monolayer. In our set-up, the monolayers are coupled to an effective surface tension of 77 mN/m, which is the sum of the cholesterol/water and water/vacuum interfaces:

$$\gamma_s = \gamma_m + \gamma_{wv} \tag{2}$$

where γ_m is the cholesterol/water interfacial tension and γ_{wv} is the surface tension at the water/vacuum interface. The value for γ_{wv} has been previously calculated by Chen et. al. [2] for the SPC water model being 55 mN/m at 288 K. Thus the surface pressure π was calculated from the surface tension through the following relation:

$$\pi = \gamma_{wv} - \gamma_m \tag{3}$$

SUPPLEMENTARY RESULTS

Dimeric conformation of βCDs in solution

To study the stability of βCD dimers in solution, simulations were performed starting from three different dimeric conformations, namely head-head, head-tail and tail-tail (Fig. S1A). The head-head conformer is formed by the hydrogen bonding of two βCD monomers trough their hydroxyl groups attached to the carbons C_2 and C_3 of the ring (see Fig. S1B for carbon numbering). The head-tail conformer is the result of the interaction of the hydroxyl groups attached to C_2 and C_2 of one ring

with the hydroxypropyl groups attached to the C_6 of the second ring. In the tail-tail case, the two rings interact with each other through their hydroxypropyl groups. Each βCD dimer was placed in the center of a cubic box of 6 nm edge and filled with 7,000 SPC water molecules, and simulated for 200 ns. Each simulation was repeated several times, using different initial velocities, to test the reproducibility of our results. Force field parameters and simulation conditions were equal to those used for the simulations in the main manuscript.

Our simulations show that the stability of the βCD dimer depends highly on the degree of the intermolecular interaction. The number of intermolecular hydrogen bonds that can be formed in the head-head orientation is larger compared to the head-tail and tail-tail orientations. In line with this criterion, at the end of the simulation, only the head-head conformation proved to be stable, keeping the initial level of inter molecular hydrogen bonds (Fig. S1C). Both head-tail and tail-tail conformers dissociate on a time scale of 1-10 ns. We conclude that the head-head orientation is the most stable state of dimerization for βCD. Therefore, the simulations described in the main manuscript were set-up using the head-head conformer.

Ineffective cholesterol extraction by βCDs

In the main manuscript it is concluded that the most efficient cholesterol extraction requires a head-head dimer adsorbed at the monolayer interface and stabilized in an upright position. To test the ability of CDs to extract cholesterol in other orientations, we performed additional MD simulations of various βCD dimeric conformations in direct contact with a cholesterol monolayer. As an example, a series of snapshots (figure S2A-C) depicts the disruption of four dimers initially placed in head-tail conformation and in direct contact with the monolayer. At the end of the 200 ns simulation the dimers are no longer stable and have dissociated completely. The monomers remain adsorbed at the monolayer surface but are not able to extract them (although cholesterol hydroxyl groups do interact with the CD interior, Fig. S2C). We observed the same effect with the tail-tail conformation. The head-head dimer, on the other hand, remains stable in its dimeric form, but prefers to tilt on the monolayer surface if it is not stabilized by neighboring CDs. This is exemplified in Figure S2D,E for the case of a single head-head dimer in direct contact with the monolayer. The tilting of the dimer prevents the extraction of cholesterol, at least on the time scale of the simulation (200 ns).

Potentials of mean force (PMFs)

Figure S3 show the detailed PMFs used to calculate the free energies of a number of important sub-steps of the cholesterol extraction process. As discussed in the main article, there is a difference of 10 kJ mol^{-1} between the association energy of cholesterol with the monomeric (A) and the dimeric form (B) of cyclodextrins. The free energy associated with the βCD dimer desorption from a cholesterol monolayer surface (C) as well as the βCD non-asisted cholesterol extraction energy from the monolayer (D) are also depicted. In Table S1 we compare the association constant for cholesterol/β-CD calculated from our simulations (eq:4) with experimental data available from the literature [3, 4, 5]. Experimentally it is difficult to distinguish between the 2:1 and 1:1 complexes, and different methods predict binding constants varying over orders of magnitude. Keeping these limitations in mind, a comparison of the binding constants calculated from our PMFs predict an order of magnitude comparable to the experimental estimate for β-CD and HP-β-CD, assuming the experiments probe the 2:1 stoichiometry. In experiments on DM-β-CD [3], the binding affinity could be differentiated between the 2:1 and 1:1 stoichiometries; the 2:1 case showed a much higher affinity in line with our results for β-CD.

References

[1] Hess B, Kutzner C, van der Spoel D, Lindahl E (2008) Gromacs 4: Algorithms for highly efficient, load-balanced, and scalable molecular simulation. J Chem Theory Comput 4: 435-447.

[2] Chen F, Smith PE (2007) Simulated surface tensions of common water models. J Chem Phys 126: 221101-221101.

[3] Nishijo J, Moriyama S, Shiota S (2003) Interactions of cholesterol with cyclodextrins in aqueous solution. Chem Pharm Bull (Tokyo) 51: 1253-1257.

[4] Frijlink HW, Eissens AC, Hefting NR, Poelstra K, Lerk CF, et al. (1991) The effect of parenterally administered cyclodextrins on cholesterol levels in the rat. Pharm Res 8: 9-16.

[5] Breslow R, Zhang B (1996) Cholesterol recognition and binding by cyclodextrin dimers. J Am Chem Soc 118: 8495-8496.
